# Changes in Quality of Life and Loneliness Among Middle-Aged and Older Adults Participating in Therapist-Guided Digital Mental Health Intervention

**DOI:** 10.3389/fpubh.2021.746904

**Published:** 2021-12-09

**Authors:** Christine E. Gould, Chalise Carlson, Ana Jessica Alfaro, Christina F. Chick, Martha L. Bruce, Valerie L. Forman-Hoffman

**Affiliations:** ^1^VA Palo Alto Health Care System, Geriatric Research, Education and Clinical Center, Palo Alto, CA, United States; ^2^Department of Psychiatry and Behavioral Sciences, Stanford University School of Medicine, Palo Alto, CA, United States; ^3^VA Palo Alto Health Care System, Mental Illness Research, Education, and Clinical Center (MIRECC), Palo Alto, CA, United States; ^4^Geisel School of Medicine at Dartmouth and Dartmouth-Hitchcock Health, Hanover, NH, United States; ^5^Meru Health, Inc., San Mateo, CA, United States

**Keywords:** aging, depression, digital health, digital therapeutics, mHealth, smartphone

## Abstract

**Background:** This study aimed to examine the effects of a 12-week multicomponent mobile app-delivered intervention, the Meru Health Program (MHP), on mental health quality of life (QoL) and loneliness among the middle-aged and older adults with depression symptoms.

**Methods:** The eligible participants (*M* age = 57.06, *SD* = 11.26 years) were enrolled in the MHP, a therapist-supported mobile intervention. Using a non-randomized pre-post design, change in mental health QoL [WHO QoL Brief (WHOQOL-BREF) psychological health] and loneliness (UCLA Loneliness Scale) from baseline to post-treatment were examined. Time of enrollment [pre- vs. post-coronavirus disease 2019 (COVID-19)] was included as a between-subjects factor in the repeated measures analyses.

**Results:** Forty-two participants enrolled prior to the COVID-19 pandemic; eight enrolled after the pandemic began. Among the pre-COVID-19 enrollees, increase in mental health QoL, *F*_(1, 38)_ = 12.61, *p* = 0.001, η^2^ = 0.25 and decreases in loneliness emerged, *F*_(1, 38)_ = 5.42, *p* = 0.025, η^2^ = 0.13. The changes in mental health QoL, but not loneliness, held for the combined sample, such as post-COVID-19 enrollees, *F*_(1, 44)_ = 6.02, *p* = 0.018, η^2^ = 0.12. The regression analyses showed that increases in mindfulness were associated with the increased mental health QoL and decreased loneliness.

**Conclusion:** Therapist-supported digital mental health interventions, such as the MHP, have the potential to improve mental health QoL and decrease loneliness among the middle-aged and older adults. The findings for loneliness may not hold during the periods of mandated isolation. Instead, therapists supporting digital interventions may need to tailor their approach to target loneliness.

## Introduction

Loneliness, a subjective feeling of social isolation, afflicts more than a third (35%) of adults aged 45 and older (1). Furthermore, loneliness co-occurs with numerous chronic health conditions (2, 3), increases risk of dementia (4), and leads to increased morbidity and mortality (5, 6). The health epidemic of loneliness continues to worsen, most recently due to acute factors, such as the [coronavirus disease 2019 (COVID-19)] pandemic that led to or exacerbated social isolation (7).

The interventions targeting loneliness primarily focus on the enhancing social skills, providing social support, increasing social access, and/or targeting maladaptive thoughts (8). The meta-analytic findings suggest that the interventions that include components to target maladaptive thoughts or social cognitions were most efficacious (8). A review focused on older adults found that most of the interventions for loneliness were delivered in a group-based format (66%) and often utilized primarily educational interventions (9). The authors concluded that the interventions that promoted social connections were most effective (9). Moreover, the use of information and communication technology (e.g., social media and email) to foster connectivity among the older adults is gaining attention. The studies have demonstrated that greater access to and use of technology among the older adults is associated with lower depression, fewer chronic conditions, and greater perceived social support, health, and subjective well-being (10, 11). These findings suggest that the use of technology in late life can alleviate loneliness as well as support mental and physical health.

Aligned with the previous findings of the importance of addressing loneliness, the potential benefits of utilizing technology to promote social connectedness, and the inherent social connectedness in group interventions, we examined whether a multicomponent digital intervention may decrease loneliness. The digital intervention [Meru Health Program (MHP)] is a therapist-supported program that incorporates mindfulness and cognitive behavioral interventions to decrease depression and anxiety. Furthermore, the MHP is delivered in a group format with opportunities for social support through the therapist-moderated discussion among the group participants. Our previous work with middle-aged and older adults has demonstrated that the 8-week MHP program led to significant reductions in anxiety and depression (12) and subjective improvements in multiple domains, such as increased activity participation and improved social interactions (13). Furthermore, other work has shown that the skills incorporated into MHP intervention, specifically mindfulness skills, are found to reduce loneliness among the younger adults (14). Additionally, brief behavioral interventions, such as behavioral activation, are shown to decrease loneliness among the home-bound older adults with depression (15, 16). Taken together, it is expected that the MHP would both increase mental health quality of life (QoL) and decrease loneliness through increasing acceptance, improving self-regard/self-compassion, reducing negative cognitions, and promoting engagement in the present moment through informal mindfulness practices (17).

The present study aimed to extend previous findings on the benefits of the MHP on reducing depressive and anxiety symptoms to two important domains: QoL and loneliness. This study had two aims, which were to examine whether participation in the MHP resulted in change in (1) mental health QoL and (2) loneliness in a sample of middle aged and older adults with the depressive symptoms. In an exploratory aim, we investigated whether change in mindfulness was associated with change in mental health QoL and loneliness.

## Methods

### Study Design

This study was a 12-week non-randomized pre-post examination of the MHP in middle-aged and older adults (Clinicaltrials.gov NCT03652948).

### Participants

Recruitment of the participants occurred between April 2019 and March 2020 and between August 2020 and October 2020. The advertisements consisted of flyers posted on public community boards, newspaper advertisements, and digital advertisements (Craigslist and Facebook) targeting people aged 40 years and older within the California Bay Area. During the COVID-19 pandemic, digital ads on Craigslist and Facebook linked to a secure online contact survey became the primary source of recruitment between August and September 2020.

The eligible participants were the residents from California, with a smartphone capable of running the MHP app, and had increased depressive symptoms defined as Patients Health Questionnaire nine-item Scores ≥7 [PHQ-9; (18)]. Exclusion criteria applied during the initial telephone screen included presence of bipolar disorder, potential psychosis assessed using the Mini Neuropsychiatric Interview 7.0.2 [MINI; (19)] psychosis screening questions, substantial alcohol use as measured by the AUDIT-C [AUDIT-C ≥ 5; (20)], possible cognitive impairment as determined by the Short-Blessed Test [SBT ≥ 6; (21)], active suicide ideation [P4 Suicide Risk Screener; (22)], and participation in ongoing psychotherapy. The participants were allowed to enroll if taking psychotropic medications as long as they were on a stable dose for >30 days.

### Measures

#### Demographics and Health Questionnaire

At baseline, the participants completed a demographics and health questionnaire that collected information about race/ethnicity, marital status, living situation, education, general health information, and eight health conditions (arthritis, asthma/bronchitis, cancer, diabetes, epilepsy, heart disease, hypertension, and stroke). Health conditions were tallied to create a variable capturing the total number of current health conditions.

#### Mini Neuropsychiatric Interview 7.0.2

At baseline, a trained study personnel completed a brief semi-structured psychiatric diagnostic interview, the MINI (19), with the participants to identify the presence of current mental health disorders.

#### Patients Health Questionnaire 9-Item

The PHQ-9 (18) is a 9-item self-report scale that assesses the frequency of depressive symptoms in the past 2 weeks using a scale from 0 (not at all) to three (nearly every day). Higher scores indicate more severe depressive symptoms. The PHQ-9 has strong evidence of internal consistency, test-retest reliability, validity, and sensitivity and specificity for detecting depression (18, 23). Furthermore, it has been shown to be sensitive in detecting symptom change (23, 24). The PHQ-9 was administered at the telephone screen, baseline, week 5, week 9, and post-treatment.

#### Cognitive and Affective Mindfulness Scale-Revised

The Cognitive and Affective Mindfulness Scale-Revised CAMS-R (25) is 10-item measure of mindfulness that assesses attention, present-focus, awareness, and acceptance. The items are scored from one (rarely/not at all) to four (almost always) with higher scores indicative of more frequent mindfulness experiences. The CAMS-R has adequate internal consistency, and strong evidence of convergent and discriminant validity (25). Further, the CAMS-R has been demonstrated to be sensitive to change in the treatment studies (26). This measure was administered at baseline, week 5, week 9, and post-treatment.

#### WHO QoL-Brief

Developed by the WHO, the WHO QoL-Brief (WHOQOL-BREF) (27), has a total of 26 items related to five domains of quality of life: overall QoL and general health, physical health, psychological health, social relationships, and environment. Each item is scored on a scale of one (very dissatisfied) to five (very satisfied), thus higher scores indicated better QoL. Our variable of interest was the psychological health subscale, which is referred to as mental health QoL herein. This subscale contains items related to body image, the frequency of positive and negative feelings, self-esteem, spirituality, and thinking abilities (e.g., learning, memory, and concentration). This measure and its subscales have demonstrated good psychometric properties, such as discriminant and content validity, internal consistency, and test-retest reliability (27). The WHOQOL-BREF was measured at baseline and post-treatment.

#### UCLA Loneliness Scale

The University of California, Los Angeles (UCLA) Loneliness Scale (version 3; 28) is a 20-item measure of subjective feelings of loneliness and social isolation. The items are scored based on the frequency with which respondents perceive each statement to be self-descriptive. The scores range from one (never) to four (often), with higher scores indicating greater loneliness. The UCLA Loneliness Scale is shown to have good psychometric properties, such as high internal consistency, test-retest reliability, convergent validity, and construct validity (28). This measure was administered at baseline and post-treatment.

### Procedures

#### Baseline Assessment

After determining eligibility through the telephone screen, the participants attended a baseline visit (in person pre-COVID-19, *n* = 42; over the telephone and *via* internet surveys after the COVID-19 pandemic began, *n* = 8). The participants completed questionnaires and partook in a semi-structured psychiatric interview (MINI). At the end of the baseline assessment, the eligible participants were provided with a brief overview of the MHP enrollment process. After the visit, a research team member referred the participant to Meru Health for MHP enrollment. The MHP therapist or clinical coordinator set up a time for the participant to have a brief intake call with the therapist. The participant completed standard questionnaires as part of the Meru Health intake process. The week prior to each group starting, the participants received an email with instructions to download the app and a unique link to sign into the app.

#### Intervention

The MHP v3.0 is 12-week mobile app-delivered intervention grounded in mindfulness and cognitive behavioral techniques. The MHP is delivered to a group of patients who work through the program as a cohort overseen by a therapist. The app delivers informational videos and guided practices that aim to help manage depression, anxiety, and burnout (stress). App content is delivered in the weekly themes that address topics, such as mindfulness, thinking traps, self-compassion, values, sleep, and nutrition. Additional program features include therapist (i.e., licensed marriage and family therapist or clinical social worker), and anonymous peer support from other group members. The therapist uses a dashboard to oversee the MHP progress of a patient. The therapist interacts with the patients by sending weekly informational emails, asynchronous secure messaging within the app, and conducting phone/video calls when needed. The peer support consists of therapist-moderated discussions that allow the group members to anonymously share thoughts and experiences with the practices. The group members can respond to each other comments by selecting from a menu of pre-determined responses (e.g., “that sounds hard).” Beyond this study, MHP is available in the United States and Finland through employer-based wellness plans, insurance, and university-based mental health clinics.

#### Interim and Post-Treatment Assessments

The PHQ-9 and CAMS-R measures were collected at weeks 5 and 9 by phone or through secure online surveys according to the preference of participants. The measures, such as PHQ-9, CAMS-R, WHOQOL-BREF, and UCLA Loneliness Scale were completed after the participants finished the 12-week MHP (in-person *n* = 29; by phone/internet surveys *n* = 17).

### Data Analyses

Descriptive statistics were utilized to describe the sample characteristics. Sample distribution kurtosis and skew were reviewed. The sample was determined to be normally distributed, so the parametric statistics were used. Using repeated measures ANOVA models, we examined change from baseline to 12 weeks for mental health QoL (WHOQOL-BREF psychological health scale) and loneliness (UCLA Loneliness Scale) for the pre-COVID-19 enrollees first. Next, these analyses were conducted with the entire sample, such as time of enrollment (pre- vs. post-COVID-19) as a between-subjects factor. Alpha was set at 0.05. The uncontrolled effect sizes using hedge's g were calculated.

Correlates of change were examined in the exploratory analyses. Change scores in mental health QoL were calculated by subtracting the baseline score from the post-treatment scores. For mental health QoL, positive change indicates improvements (i.e., increase in scores), whereas for UCLA Loneliness scores, negative change indicates decrease in loneliness (i.e., decline in scores). First, single-sample *t*-tests assessed whether the changes were significantly different from zero. Second, the linear regression analyses examined whether the baseline variables were associated with change in the dependent variables (mental health QoL and loneliness). Third, the linear regression analyses examined whether change in depression (PHQ-9) or mindfulness (CAMS-R) were associated with change in dependent variables (mental health QoL and loneliness).

## Results

### Participant Flow and Characteristics

**Figure 1:**
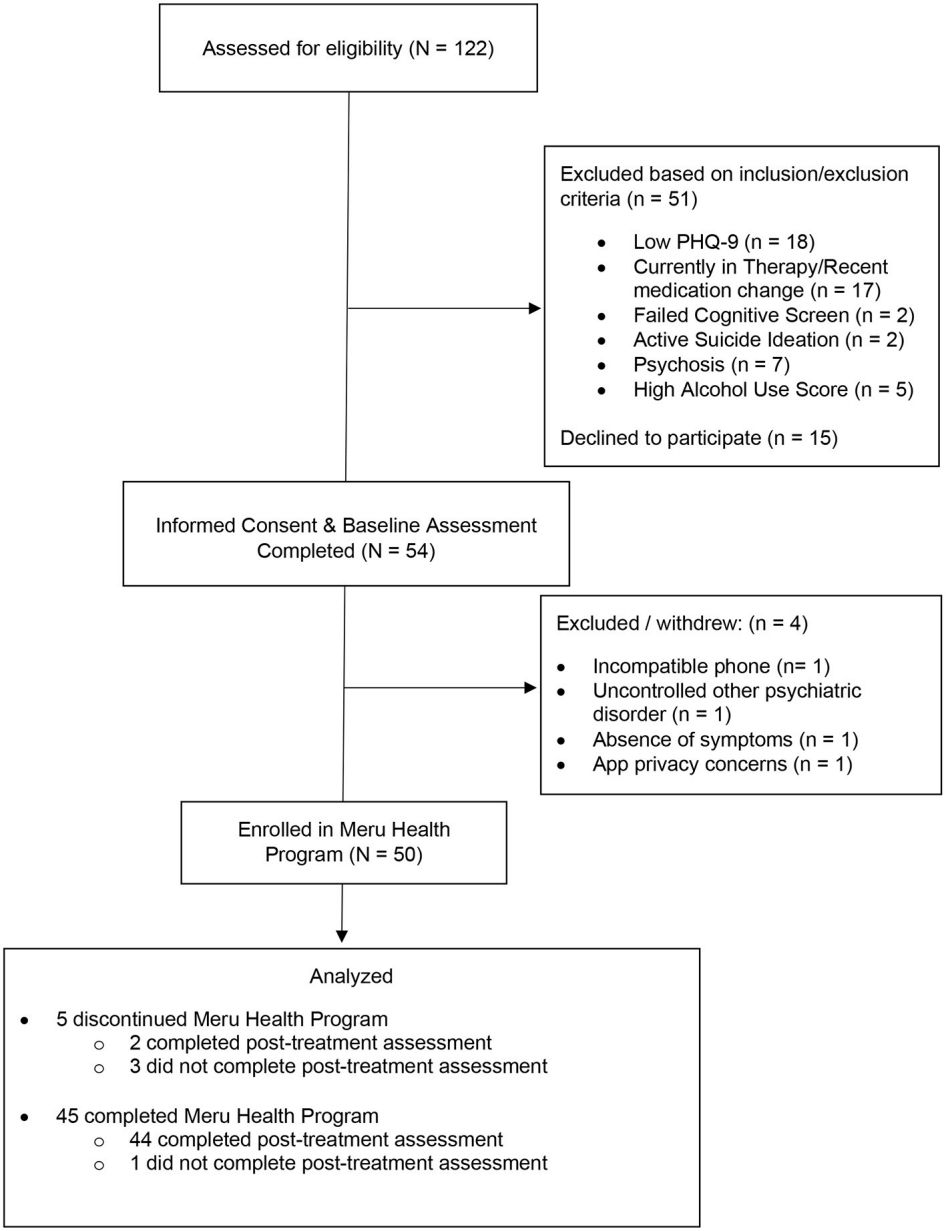
Flow of the assessed and enrolled participants (*N* = 50).

Fifty-four participants completed the baseline assessments; two were excluded (due to ineligibility) and two withdrew (due to improved symptoms and privacy concerns) prior to their MHP group start date (as shown in Figure 1). Thus, 50 participants with a mean age of 57.06 (*SD* = 11.26; range: 40–81 years) were enrolled in the MHP. About 60% (*n* = 30) of enrollees were female and the majority were white, non-Hispanic (58%, *n* = 29), followed by Asian (16%, *n* = 8), and Black/African American individuals (10%, *n* = 5). Mean baseline PHQ-9 scores were 12.28 (*SD* = 5.47) and fell in the moderate depressive symptom range (10–14). Regarding the baseline psychiatric diagnoses, two-thirds (66.7%) had either current major depressive disorder (28%; *n* = 14), an anxiety disorder (16%; *n* = 8), or both major depressive disorder and an anxiety disorder (26%; *n* = 13). Among the remaining third of participants, 19% (*n* = 8) had another current psychiatric disorder and 14% (*n* = 7) did not have any current psychiatric disorders. Table 1 displays the participant characteristics at baseline.

**Table 1:** The participant characteristics (*n* = 50).

**Participant characteristics**	***N* (%)**	**M (SD)**
Age (years)		57.1 (11.3)
Education (years)		16.1 (2.9)
**Sex**		
Female
Male	30 (60.0%) 20 (40.0%)	
**Race/Ethnicity**		
Any Race, Hispanic
Asian
Black
White, Non-Hispanic
Other	3 (6.0%) 8 (16.0%) 5 (10.0%) 29 (58.0%) 4 (8.0%)	
**Marital status**		
Single
Married
Separated/Divorced
Widowed	17 (34.0%) 18 (36.0%) 13 (26.0%) 2 (4.0%)	
**Employment**		
Full-time
Part-time
Unemployed
Retired
Other	12 (24.0%) 13 (26.0%) 12 (24.0%) 12 (24.0%) 1 (2.0%)	
**Number health conditions**		
0
1
2
3 or more	18 (36.0%) 10 (20.0%) 5 (10.0%) 17 (34.0%)	
**Prevalence of current psychiatric diagnoses^a^**		
Major Depressive Disorder
Anxiety Disorder(s)
Posttraumatic Stress Disorder
Binge Eating Disorder	27 (54.0%) 21 (42.0%) 8 (16.0%) 7 (14.0%)	
PHQ-9		12.3 (5.5)
UCLA Loneliness Scale		50.9 (10.9)
**WHOQOL-BREF**		
Overall QoL and General Health
Physical Health
Psychological
Social Relationships
Environment	5.9 (2.0)
21.5 (5.1)
17.2 (4.0) 8.5 (2.7) 28.0 (5.7)	

a *Total does not equal 100% due to multiple participants exhibiting multiple diagnoses. Other psychiatric disorders account for <10%. Data shown are collapsed across the pre-coronavirus disease 2019 (COVID-19) and post-COVID enrollees*.

Data collection and enrollment intersected with the historical event of the onset of the COVID-19 pandemic, which brought about shelter-in-place restrictions. Forty-two participants enrolled prior to the COVID-19 pandemic; eight enrolled after the pandemic had begun. The pre-COVID-19 enrollees were more likely to be married or partnered [χ^2^(50) = 5.36, *p* = 0.021], have lower baseline depression scores [*t*_(48)_ = −2.10, *p* = 0.041], and have higher baseline QoL-environment subscale scores [*t*_(48)_ = 2.29, *p* = 0.027], than post-COVID-19 enrollees. No baseline differences in mental health QoL or loneliness emerged.

Of 50 enrollees, 45 (90%) completed the 12-week MHP. Reasons for discontinuation of MHP participation were financial insecurity (*n* = 1), not liking the app (*n* = 1), work responsibilities (*n* = 1), and lost to follow-up (*n* = 2). Post-treatment data was obtained for 46 participants (92%). One completer did not provide a final assessment; two non-completers did provide a final assessment. The four participants who did not complete a post-treatment assessment (one completer; three non-completers) did not differ from the 46 participants included in the analyses on demographic characteristics or baseline depression symptoms, mental health QoL, or loneliness.

###  Change in Mental Health QoL

**Figure 2:**
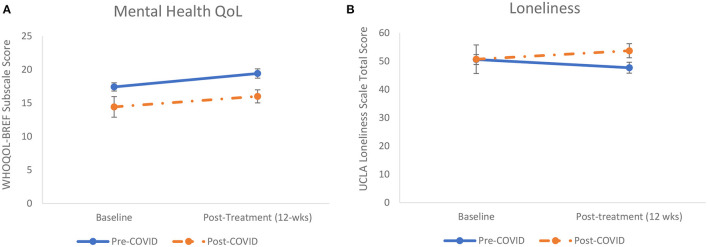
**(A,B)**. Increases in mental health quality of life (QoL) and declines in loneliness found from baseline to post-treatment (*N* = 46). **(A)** The increase in WHO QoL Brief (WHOQOL-BREF) scores represent significantly improved mental health QoL for pre-coronavirus disease 2019 (COVID-19) (solid line) and post-COVID-19 enrollees (dashed line). **(B)** The decline in UCLA Loneliness Scale scores represent significant decrease in loneliness for the pre-COVID enrollees (solid line). The dashed line shows a non-significant increase in loneliness for the post-COVID-19 enrollees. The error bars represent SEM.

A repeated measures ANOVA examined change in mental health QoL among the participants who enrolled in the MHP pre-COVID-19. Significant increase in mental health QoL as measured with the WHOQOL-BREF psychological health subscale were found with a significant main effect of time, *F*_(1, 38)_ = 12.61, *p* = 0.001, η^2^ = 0.25. The uncontrolled effect size estimated using hedge's *g* was 0.44. A second repeated measures ANOVA was conducted for the combined sample that included a variable to compare pre-COVID-19 enrollees and post-COVID-19 enrollees as a between-subjects factor. In this analysis with the combined sample, the increases in mental health QoL held, with a significant main effect of time, *F*_(1, 44)_ = 6.02, *p* = 0.018, η^2^ = 0.12. Additionally, the main effect of enrollment was significant [*F*_(1, 44)_ = 4.70, *p* = 0.036, η^2^ = 0.10], which demonstrated that the mental health QoL for the pre-COVID-19 enrollees was higher than that of post-COVID-19 enrollees (as shown in Figure 2A). The interaction of enrollment and time was not significant [*F*_(1, 44)_ = 0.09, *p* = 0.770, η^2^ = 0.002], suggesting that the increased mental health QoL were similar for pre- and post-COVID-19 enrollees.

### Change in Loneliness

A repeated measures ANOVA examined change in loneliness among the participants who enrolled in MHP pre-COVID-19. A main effect of time emerged, demonstrating significant decreases in loneliness, *F*_(1, 38)_ = 5.42, *p* = 0.025, η^2^ = 0.13. The uncontrolled effect size estimated using hedge's *g* was −0.24. A second analysis conducted with the combined sample of participants enrolled pre- and post-COVID-19 found no significant main effects for time [*F*_(1, 44)_ = 0.001, *p* = 0.977, η^2^ = 0.00] or enrollment [*F*_(1, 44)_ = 0.49, *p* = 0.486, η^2^ = 0.01]. Moreover, the interaction was not significant, [*F*_(1, 44)_ = 2.88, *p* = 0.097, η^2^ = 0.06). As seen in Figure 2B, it appears that non-significant increases in loneliness occurred for the participants enrolled post-COVID-19.

### Examining Correlates of Change

The initial step in examining correlates of change was to determine whether the change scores significantly differed from zero. For the pre-COVID-19 sample, change was significantly different from zero for both mental health QoL [*t*_(38)_ = 3.55, *p* = 0.001] and for loneliness [*t*_(38)_ = −2.33, *p* = 0.025). When including the post-COVID-19 enrollees, only mental health QoL was significantly different from zero. Consequently, the linear regression analyses focused on the pre-COVID-19 sample that displayed significant change in the dependent variables from baseline to post-treatment. Next, we conducted a regression model that included the baseline factors (i.e., PHQ-9 scores, current depression and anxiety diagnoses, gender, and age) as the independent variables. This analysis was not significant, indicating that the baseline factors were not significant correlates of change in QoL or loneliness (results not shown). Then, we examined whether change in the depression symptoms (PHQ-9) and in mindfulness (CAMS-R) from baseline to post-treatment were predictors of change in QoL and in loneliness. As displayed in Table 2, the regression analyses demonstrated that mindfulness had a positive association with improved mental health QoL (B = 0.43, *p* = 0.004) and a negative association with loneliness (B = −0.40, *p* = 0.007). Change in the depressive symptoms had a negative association with mental health QoL that approached significance (B = −0.29, *p* = 0.048). Change in the depressive symptoms were significantly and positively associated with loneliness (B = 0.34, *p* = 0.019).

**Table 2:** The regression models examining correlates of change for pre-COVID-19 enrollees (*N* = 38).

**Correlate**	**b (SE)**	**b 95% CI**	**Beta**	***t***	***p***	***R* ^**2**^**	**Fit**
**Model examining change in mental health quality of life**							
(Intercept)	0.16 (0.65)						
PHQ-9 Change	−0.22 (0.11)	−0.44, −0.002	−0.29	−2.05	0.048		
CAMS-R Change	0.33 (0.11)	0.11, 0.55	0.43	3.06	0.004		
						0.36	*F*_(2, 38)_ = 9.96, *p* <0.001
**Model examining change in loneliness**							
(Intercept)	1.40 (1.43)						
PHQ-9 Change	0.58 (0.24)	0.10, 1.07	0.34	2.45	0.019		
CAMS-R Change	−0.68 (0.23)	−1.16, −0.19	−0.40	−2.84	0.007		
						0.37	*F*_(2, 38)_ = 10.49, *p* <0.001

*Two regression analyses are displayed. Higher scores on the mental health quality of life (QoL) measure indicate better QoL. In contrast, higher loneliness scores indicate more loneliness*.

## Discussion

These findings show that the MHP, a therapist-supported digital mental health intervention, was associated with increased mental health QoL and decreased loneliness among the middle-aged and older adults in a non-randomized pre-post study. Notably, the improvements in mindfulness across treatment were associated with increased mental health QoL and decreased loneliness. In contrast, the declines in depression symptoms only corresponded to the declines in loneliness. Taken together, these findings suggest that one mechanism through which the MHP may impact loneliness and mental health QoL is by improving mindfulness. The finding that the mindfulness component of this intervention may reduce the subjective experience of loneliness dovetails with findings of a recent dismantling study that demonstrated that the combination of present focus and acceptance skills from mindfulness resulted in the declines in loneliness compared with present focus alone (13). Nonjudgment and acceptance that is trained through mindfulness practice may help alleviate maladaptive thought patterns and emotions that accompany loneliness.

Although the future controlled studies need to replicate these findings, this investigation extends prior findings that support the reductions in psychiatric symptoms (12, 29–31) to QoL and loneliness. The improvement in mental health QoL likely correspond to the components of the MHP curriculum that target not only depression and anxiety, but also address the sleep difficulties and other mental health-related topics, such as relationships, self-compassion, values, and eating habits. Inclusion of therapist support likely contributes to the low dropout rate found in this study compared with unsupported digital interventions, which have higher drop-out rates and small treatment effects (32).

Loneliness negatively impacts physical, cognitive, and mental health and longevity, particularly among the middle-aged and older adults (2–6). Thus, the declines in loneliness following participation in a therapist-supported digital mental health intervention hold promise. Leveraging digital mental health interventions may be a critical step in increasing access to the efficacious interventions that older adults otherwise would not access due to scarcity of trained geriatric mental health providers (33). Further research is needed to examine the MHP and similar interventions in the controlled studies to better understand variations of intervention outcomes by age.

One important caveat to these findings is that when a historical event (COVID-19 pandemic) occurred, initiating shelter-in-place requirements for many participants, the decline in loneliness no longer held. The increase in mental health QoL remained significant regardless of shelter-in-place status, which highlights the potential benefits of using the digital mental health interventions for the middle-aged and older adults. It is possible that restricting opportunities to socialize in person cannot be overcome by the digital mental health interventions that otherwise have the potential to decrease loneliness. Therapists supporting the mobile health (mHealth) interventions may need to tailor their approach during the COVID-19 pandemic by encouraging the patients to make video calls and explore possibilities of online interest groups and virtual meet-ups in order to mitigate loneliness. The group support aspect of the MHP has the potential to help participants with the feelings of loneliness and subjective isolation in their mental health-related struggles; however, our previous work has demonstrated that this component was deemed to be less helpful than the information provided within the app, the daily practices, and support of the therapists (12). In contrast, for the younger users, use of the group support feature in MHP predicted the declines in depression scores (29). It is possible that using preset responses for group members, while important from a safety, risk reduction, and confidentiality standpoint, may limit engagement with this feature and reduce the usefulness of the group support aspect of the intervention for the older users in particular. Further consideration into incorporating the meaningful peer interactions in mHealth interventions targeting older users is needed.

Several limitations should be noted. First, this study lacked a control condition, thereby preventing any analyses or conclusions regarding the effect of the MHP on mental health QoL or loneliness compared with other interventions. As our study was not a dismantling study, it is possible that other MHP intervention components, such as behavioral activation, could have led to these improvements as well. Second, our findings are limited by the small number of enrollees after the COVID-19 shelter-in-place restrictions were enacted. Third, a measurement of objective social isolation was not obtained, thus limiting the interpretation of the findings, particularly with regard to the differences among the pre-COVID-19 and post-COVID-19 enrollees. Fourth, the sample was relatively homogeneous, consisting of white, non-Hispanic individuals, thus limiting information that can be gleaned about the effects of MHP among other groups. Fifth, our study was limited to the participants who had access to a smartphone, thus potentially limiting generalizability of the findings to those with higher technology proficiency and higher socioeconomic status (i.e., those who could afford such a device).

Despite these limitations, this investigation provides preliminary support for the effect of a therapist-supported mHealth intervention on yielding mental-health related QoL benefits, such as reduced loneliness. The regression analyses suggest that the components of MHP targeting mindfulness may be particularly important in yielding these mental health benefits. This study further demonstrates that older adults may benefit from the digital mental health interventions, which may, in turn, increase their access to mental healthcare. Future studies are needed to examine whether tailored therapist support targeting loneliness may enhance the effects of digital mental health interventions on this growing problem.

## Data Availability Statement

The datasets presented in this article are not readily available because data will be available upon reasonable request with agreement from funder. Requests to access the datasets should be directed to Christine Gould, cegould@stanford.edu.

## Ethics Statement

The studies involving human participants were reviewed and approved by Stanford University Institutional Review Board. The patients/participants provided their written informed consent to participate in this study. During the COVID-19 pandemic, participants provided oral consent upon reviewing an informed consent document.

## Author Contributions

CG conceptualized the research question, designed the study, oversaw data collection, conducted analyses, and drafted the manuscript. CCa collected data and assisted with drafting of the manuscript. AA assisted with drafting the manuscript. CCh assisted with data collection and with editing the manuscript. MB and VF-H edited the manuscript. All authors contributed to the article and approved the submitted version.

## Funding

Meru Health, Inc provided funding for this project. CG is supported by a Career Development Award (IK2 RX001478) from the Department of Veterans Affairs Rehabilitation Research and Development Service. The Stanford REDCap platform is developed and operated by the Stanford Medicine Research IT team. The REDCap platform services at Stanford are subsidized by (a) the Stanford School of Medicine Research Office and (b) the National Center for Research Resources and the National Center for Advancing Translational Sciences, National Institutes of Health, and through grant UL1 TR001085.

## Author Disclaimer

Views expressed in this article are those of the authors and not necessarily those for the Department of Veterans Affairs or the Federal Government.

## Conflict of Interest

The study received funding from Meru Health, Inc. The funder had no role in study design, data collection and analysis, or decision to publish. The funder's Chief Research Officer VF-H was a co-author on the study and assisted with editing the manuscript. VF-H receives salary from the company and owns options of the company. The remaining authors declare that the research was conducted in the absence of any commercial or financial relationships that could be construed as a potential conflict of interest.

## Publisher's Note

All claims expressed in this article are solely those of the authors and do not necessarily represent those of their affiliated organizations, or those of the publisher, the editors and the reviewers. Any product that may be evaluated in this article, or claim that may be made by its manufacturer, is not guaranteed or endorsed by the publisher.
